# Anaemia among Patients of Heart Failure in a Tertiary Care Centre of Nepal: A Descriptive Cross-sectional Study

**DOI:** 10.31729/jnma.6486

**Published:** 2021-09-30

**Authors:** Abhishek Bhandari, Prashant Shah, Naveen Kumar Pandey, Richa Nepal, Ojaswee Sherchand

**Affiliations:** 1 Department of Internal Medicine, B.P. Koirala Institute of Health Sciences, Dharan, Nepal; 2 Department of Biochemistry, B.P. Koirala Institute of Health Sciences, Dharan, Nepal

**Keywords:** *anaemia*, *heart failure*, *iron deficiency*, *prevalence*

## Abstract

**Introduction:**

Anaemia is an important comorbidity common in patients with heart failure and is associated with poor clinical status and worse outcomes. In Nepal few studies have evaluated anaemia amongst patients suffering from heart failure. We intended to find out the prevalence of anaemia in patients with heart failure in a tertiary care centre.

**Methods:**

This is a descriptive cross-sectional study conducted among patients of heart failure presenting to tertiary care hospital in eastern Nepal from April 2017 to January 2018. Ethical approval was taken from the Institutional Review Committee of a tertiary care centre (reference number: IRC/0842/016). Using the convenience sampling method, 100 patients were enrolled in the study. Blood samples from the patients were taken for haemoglobin and serum iron studies. Data was analysed using Statistical Package for Social Sciences version 11. Point estimate at 95% Confidence Interval was calculated, with frequency and percentage.

**Results:**

Among 100 patients with heart failure, 82 (82%) (74.47-89.53 at 95% Confidence Interval) had anaemia. Mean haemoglobin level of the study population was 10.40±2.73g/dl. Fifty four (54%) of patients had iron deficiency status irrespective of presence or absence of anaemia.

**Conclusions:**

Prevalence of anaemia among patients of heart failure in our study was found to be higher than various other homologous international studies.

## INTRODUCTION

Heart failure is a symptom complex that co-exists with various comorbidities like Chronic Kidney Disease (CKD), anaemia, diabetes mellitus, systemic hypertension etc. that contribute to increased hospitalization, morbidity and mortality.^1^ Anaemia with or without iron deficiency is a proven comorbid condition associated with heart failure that increases morbidity and mortality, and impairs quality of life.

Observational studies have shown that prevalence of anaemia among patients of heart failure ranges from 40-60%.^2-5^ Intriguingly, iron deficiency with or without anaemia in heart failure has been related to disease severity and greater predictive value for outcome than the presence of anaemia.^3^ Data related to anaemia and iron deficiency in patients with heart failure is very limited in Nepalese setup.

This research aims to find out the prevalence of anaemia in patients with heart failure presented to a tertiary care center in eastern Nepal.

## METHODS

This was a descriptive cross-sectional study conducted from 4^th^ April 2017 to 31^st^ January 2018. The study was conducted at B.P. Koirala Institute of Health Sciences (BPKIHS) which is a tertiary care centre located in eastern Nepal. The Institutional Review Committee (IRC) of BPKIHS had approved our research protocol prior to the start of the study (reference number: IRC/0842/016). All the patients enrolled in the study gave prior written informed consent for participation in the study meaning they were subjected to history taking, physical examination and appropriate lab investigations. Patients coming to the medical outpatient rooms, admitted to medical wards and coronary care units with a diagnosis of heart failure were included in the study. Patients who were on iron supplementations or blood transfusion were excluded. The patients who were already diagnosed with other conditions causing anaemia that were uncorrected or under treatment were also excluded. Those conditions included coexisting chronic kidney disease, bleeding peptic ulcers, chronic liver disease, connective tissue disease and any chronic malignancy. Pregnant patients were also excluded. All the consecutive patients diagnosed of Heart failure meeting the inclusion criteria were included in the study till the sample size was met.

The sample size was calculated using the formula

n = Z^2^ × p × q / e^2^

  = Z^2^ × p × (1-p) / e^2^

  = (1.96)^2^ × 0.37 × (1-0.37) / (0.1)^2^

  = 89.5

Where,

n = required sample size
Z = 1.96 at 95% Confidence Interval (CI)
p = prevalence of anaemia in patients with heart failure in reference population^6^
e = margin of error, 10% in this study.

Hence, the calculated sample size was 89.5. Taking a 10% non-response rate and rounding the figure, we included 100 patients in the study.

Data was collected on a structured questionnaire, which included questions on demographic variables and relevant clinical history. Detailed clinical examination was done and diagnosis of Heart failure was made based on Framingham diagnostic criteria for heart failure at the time of presentation.^7^ Blood samples for all relevant investigations except for serum iron studies were drawn on the day of presentation to hospital. Blood for serum iron studies were drawn 2 days after admission in case of admitted patients and on day of presentation in case of patients visiting outpatient rooms. Patient also underwent 1 2 lead electrocardiograms and 2-dimensional echocardiography for the assessment of cardiac anatomy and function.

Laboratory estimation of haemoglobin was done via automated analyser from 2 ml of whole blood of patient mixed with ethylene di-amine tetra acetic acid. Similarly, serum iron and unsaturated iron binding capacity (UIBC) were measured via colorimetric assay using ascorbate and ferrozinc with 2-3 ml of patients' serum/plasma free from haemolysis respectively.

Serum Ferritin was measured via Particle enhanced immunoturbidimetric assay using 2-3 ml of serum/plasma. Serum iron studies were done using Cobas c311 autoanalyzer (Roche Diagnostics) in the Department of Biochemistry, BPKIHS. We used World Health Organization's (WHO) definition of anaemia wherein haemoglobin less than 13g/dl in men and less than 1 2 g/dl in non-pregnant women was considered anaemia. For defining iron deficiency in patients with heart failure, we used the criteria used in Ferinject® Assessment in patients with IRon deficiency and chronic Heart Failure (FAIR-HF) trial wherein absolute iron deficiency to be considered when serum ferritin was <100 ng/ml and relative iron deficiency to be considered when transferrin saturation was <20% with ferritin between 100-299 ng/ml.^8^

Data was entered in Microsoft Excel sheet. Error and inconsistency was verified after checking the source document (questionnaire). IBM Statistical Package for the Social Sciences version 1 1 was used to analyse the data. Descriptive statistics were presented with frequencies and percentages for categorical variables. Point estimate at 95% Confidence Interval was calculated, with frequency and percentage.

## RESULTS

**Figure 1:**
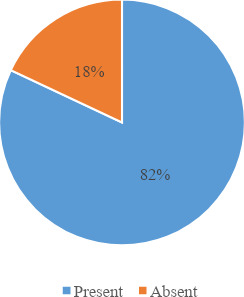
Prevalence of anaemia in patients of heart failure (n=100).

A total of 100 patients of heart failure presenting to the medical outpatient rooms, wards and coronary care unit were enrolled in the study. A total of 82 (82%) (74.4789.53 at 95% Confidence Interval) had anaemia in our study (Figure 1).

A total of 54 (54%) patients had presence of iron deficiency irrespective of the presence or absence of anaemia with 34 (34%) and 20 (20%) having absolute and relative iron deficiency respectively (Table 1).

**Table 1:** Iron deficiency status in patients with heart failure (n = 100).

Iron Deficiency Category		Frequency n (%)		
		Anaemia (n= 84)	No Anaemia (n = 16)	Total (N = 100)
No Iron Deficiency (Transferrin saturation ≥20% and serum ferritin ≥100ng/ml)		34 (34)	12 (12)	46 (46)
Iron Deficiency	Relative Iron Deficiency (Transferrin saturation <20% when serum ferritin 100-299ng/ml)	17 (17)	3 (3)	20 (20)
	Absolute Iron Deficiency (Serum Ferritin < 100 ng/ml)	31 (31)	3 (3)	34 (34)

The important baseline characteristics of patients with heart failure with and without anaemia has been illustrated below in the table. The mean age of the patients was 61.6±17.3 years and the mean haemoglobin level of the study population was 10.40±2.73 g/dl. Fifty two (52%) of patients were ≥ 65 years of age with 44 (44%) anaemic among them compared to 38 (38%) out of 48 (48%) in patients of age < 65 years. Among 51 (51%) of females, 41 (41%) had anaemia compared to 41 (41%) out of 49 (49%) of total males. 29 (29%) patients had systemic hypertension with 26 (26%) being anaemic and 31 (31%) had Type 2 diabetes mellitus with 27 (27%) being anaemic among them. Most of the patients presented in New York Heart Association (NYHA) class III and IV i.e. 85 (85%) at the time of presentation out of which 69 (69%) were anaemic. Commonest etiology of heart failure was dilated cardiomyopathy with 65 (65%) patients presenting with it with 53 (53%) anaemic among them while 29 (29%) of patients out or 35 (35%) of other etiologies of heart failure were anaemic. Fifty five (55%) of patients had severe left ventricular systolic dysfunction with 44 (44%) of anaemic among them compared to 38 (38%) anaemic out of 45 (45%) in patient with normal to moderate Left Ventricular (LV) systolic dysfunction. Seventy six (76%) patients had reduced LV ejection fraction on echocardiographic analysis with 62 (62%) anaemic among them compared to 20 (20%) anaemic among 24 (24%) of patient with preserved or borderline LV ejection fraction. Thirty (30%) out of 33 (33%) patients of atrial fibrillation had anaemia at time of presentation (Table 2).

**Table 2:** Baseline characteristics with Anaemia of patients with Heart Failure (n = 100).

Baseline Characteristics		Anaemia n (%)		Total n (%)
		Present	Absent	
Age Group (years)	< 65 years	38 (38)	10 (10)	48 (48)
	≥ 65 years	44 (44)	8 (8)	52 (52)
Gender	Male	41 (41)	8 (8)	49 (49)
	Female	41 (41)	10 (10)	51 (51)
Systemic Hypertension	Present	26 (26)	3 (3)	29 (29)
	Absent	56 (56)	15 (15)	71 (71)
Type 2 Diabetes Mellitus	Present	27 (27)	4 (4)	31 (31)
	Absent	55 (55)	14 (14)	69 (69)
NYHA^#^ Functional Class	III and IV	69 (69)	16 (16)	85 (85)
	I and II	13 (13)	2 (2)	15 (15)
Etiology of Heart Failure	Dilated Cardiomyopathy	53 (53)	12 (12)	65 (65)
	Others	29 (29)	6 (6)	35 (35)
LV^*^ Systolic Dysfunction	Severe	44 (44)	11 (11)	55 (55)
	Normal to Moderate	38 (38)	7 (7)	45 (45)
Types of Heart Failure	^$^HFrEF (EF:^Ω^ ≤40%)	62 (62)	14 (14)	76 (76)
	HF Boarderline or Preserved EF	20 (20)	4 (4)	24 (24)
Atrial fibrillation	Present	30 (30)	3 (3)	33 (33)
	Absent	52 (52)	15 (15)	67 (67)

# NYHA- New York Heart Association

* LV- Left Ventricle

$ HFrEF- Heart Failure with reduced Ejection Fraction

Ω EF- Ejection Fraction

## DISCUSSION

Anaemia is one of the commonest clinical entity worldwide with estimated global prevalence of 24.8%.^9^ It is encountered in day to day clinical practice being either the cause of presentation or one of a comorbid condition associated with another systemic disease like heart failure (H F), chronic kid ney d is ease, chronic liver disease etc. Anaemia is one of the commonest comorbidities present in patients with heart failure leading to increased morbidity and mortality. The Heart Failure Pilot Survey done by the European Society of Cardiology (ESC) in Europe in outpatient heart failure patients showed anaemia was significantly related to all-cause mortality and was also independently associated with heart failure hospitalizations, and 37% of all-cause mortality.^1^ Similarly, a meta-analysis of 34 studies conducted in patients of heart failure across the world reported crude mortality risk of anaemia was of odds ratio 1.96.^6^ However, result of a significant trial RED-HF trial (Reduction of Events With Darbepoetin Alfa in Heart Failure) suggested that anaemia by itself is probably not a mediator of poor outcome but rather marker of heart failure severity resulting in increasing hospitalizations, morbidity and mortality.^10^

Various cross-sectional studies across the world demonstrated prevalence of anaemia among patients with heart failure ranging from 30% to 60%. The study done by ESC reported above showed the prevalence of anaemia was 29%.^1^ In a meta-analysis of a total of 153,180 patients with heart failure across 34 published studies over the world, the prevalence of anaemia was reported to be 37.2%.^6^ Another hospital based study done in Tanzania in 401 patients with heart failure, the prevalence of anaemia was 57%.^4^ Our study showed that anaemia is present in 82% of patients which adds to the evidence that anaemia is a common prevalent condition in patients with heart failure.

One of the major causes of anaemia in heart failure is iron deficiency anaemia. In fact, iron studies of heart failure in various studies have shown that iron deficiency is present even when there is no overt anaemia. A study from France found that among non-anaemic patients of acute decompensated heart failure, the prevalence of iron deficiency was 57% in men and 79% in women.^2^ In another European study, iron deficiency was found to be common (50%) in patients with heart failure and was related to disease severity; moreover, the presence of iron deficiency had a greater predictive value for outcome than the presence of anaemia.^3^ Similar observation have been found in study from Tanzania where the overall prevalence of Iron deficiency was 49% distributed as 69% versus 21% in subjects with and without anaemia.^4^ Higher prevalence of iron deficiency anaemia has been attributed to various causative theories in patients with heart failure like right ventricular failure with gastrointestinal edema resulting in poor iron absorption^11^; proinflammatory cytokine activation resulting in inadequate erythropoietin production^12^; defective iron utilization despite adequate iron stores due to iron trapping in reticuloendothelial system resulting in functional iron deficiency^12^; reduced iron intake due to poor diet and gastrointestinal blood loss due to gastritis, use of platelet inhibitors and anticoagulants.^13^ Chronic iron deficiency in myocardium by itself, impairs oxidative metabolism, cellular energetics, and immune mechanisms that can cause structural and functional change in the myocardium, decreasing oxygen storage in myoglobin and reducing tissue oxidative capacity, leading to mitochondrial and LV dysfunction.^14-15^ This pathophysiological finding is further supported by treatment of iron deficiency with supplemental intravenous iron showing promising results of improving symptoms, functional NYHA class and quality of life.^16-17^ Our study showed the prevalence iron deficiency to be 54% which adds to the evidence of iron deficiency status in Nepalese patients.

On stratifying presence of anaemia to some prespecified demographics and comorbidities related to heart failure, our study showed a good proportion of patient with anaemia in groups of patients with age ≥65 years, systemic hypertension, type 2 diabetes mellitus, severe LV systolic dysfunction, heart failure with reduced ejection fraction and presence of atrial fibrillation. These findings are also supported by various international studies with some having mixed findings. In the study of patients with heart failure done by ESC in Europe, co-morbidities including anaemia were independently associated with higher age, higher NYHA functional class, ischemic etiology of Heart Failure, higher heart rate, history of hypertension, and Atrial fibrillation.^1^ In a French study, diabetes was independently associated with iron deficiency in patients with heart failure^2^; and a study form Denmark also showed higher prevalence of iron deficiency in NYHA III and IV class but no association was found between atrial fibrillation and iron deficiency.^5^ The Tanzanian study showed higher prevalence of anaemia and iron deficiency in patients with LV ejection fraction <45%.^4^ In another international pooled study, independent predictors of iron deficiency were higher NYHA class and female sex.^3^ Relationship with LV systolic dysfunction is mixed with some studies showing anaemic subjects had a better LV ejection fraction and haemoglobin is inversely related to LVEF and an increase in haemoglobin over time is associated with a decrease, not an increase, in LVEF.^18^ Association with NYHA severity might be the result of iron deficiency or anaemia rather than causal.

However, our study showed increased prevalance of anaemia among NYHA class III and IV patients than I and II, probably owing to a small number of patients with very skewed data in class I and II. The age and gender based variations in anaemia and iron deficiency status differs among various regions of the world and according to dietary habits too.

Prevalence of anaemia (82%) in Nepalese patients is alarmingly higher than that of various international studies. We are not sure whether such high prevalence of anaemia in our patients is a real picture or whether we have significant prevalence of anaemia in the general population too. Various observational studies done in communities in Nepal to assess the prevalence of anaemia in general population have been focussed in certain age groups like adolescent or non-pregnant females of certain localities of Nepal with prevalence ranging from 10-65%.^19-21^ Nevertheless, our findings recommend the need of every patient of heart failure to undergo serum iron profile estimation after management of acute decongestion and complete anaemia workup if present and intervene with intravenous iron therapy as early as possible for improving the symptoms, functional class and quality of life of patients.

Major limitation in our study was sampling bias as it was a hospital based study and most of our patients were of acute decompensated heart failure with compromised LV function because patients with NYHA class I and II usually do not present to the hospital even in regular follow up unless new clinical symptoms arise. Similarly, various inflammatory pathologies that can cause acute decompensation in heart failure can also falsely raise serum ferritin which will not show the real picture of iron deficiency. Although we analysed the blood iron profile of acutely decompensated patients 2 days after admission, ferritin could still be falsely high owing to the ongoing acute inflammatory state of acute decompensation. Thus to get a better picture of the prevalence of anaemia and iron deficiency attributable to the clinical conditions of heart failure, large scale community based case control studies are warranted so that real picture of prevalence is seen and possible association and causality is proved in Nepalese population.

## CONCLUSIONS

Prevalence of anaemia among patients of heart failure in our study was found to be much higher than various other homologous international studies. Association of anaemia with or without iron deficiency status to various coexisting clinical parameters and coexisting comorbidities of heart failure needs to be tested in well powered trials to establish causality in our settings. Anaemia in heart failure is correctable and timely correction improves their functional status and quality of life and decreases morbidity and mortality. Hence, evaluation and treatment of anaemia should be a routine in all heart failure clinics.
